# Increasing *Pneumocystis* Pneumonia, England, UK, 2000–2010

**DOI:** 10.3201/eid1903.121151

**Published:** 2013-03

**Authors:** Rishma Maini, Katherine L. Henderson, Elizabeth A. Sheridan, Theresa Lamagni, Gordon Nichols, Valerie Delpech, Nick Phin

**Affiliations:** Author affiliations: Health Protection Agency, London, UK (R. Maini, K.L. Henderson, E.A. Sheridan, T. Lamagni, G. Nichols, V. Delpech, N. Phin);; University of Chester, Chester, UK (N. Phin)

**Keywords:** *Pneumocystis jirovecii*, England, surveillance, pneumonia, *P. jirovecii* pneumonia, fungi, respiratory infections

## Abstract

After an increase in the number of reported cases of *Pneumocystis jirovecii* pneumonia in England, we investigated data from 2000–2010 to verify the increase. We analyzed national databases for microbiological and clinical diagnoses of *P. jirovecii* pneumonia and associated deaths. We found that laboratory-confirmed cases in England had increased an average of 7% per year and that death certifications and hospital admissions also increased. Hospital admissions indicated increased *P. jirovecii* pneumonia diagnoses among patients not infected with HIV, particularly among those who had received a transplant or had a hematologic malignancy. A new risk was identified: preexisting lung disease. Infection rates among HIV-positive adults decreased. The results confirm that diagnoses of potentially preventable *P. jirovecii* pneumonia among persons outside the known risk group of persons with HIV infection have increased. This finding warrants further characterization of risk groups and a review of *P. jirovecii* pneumonia prevention strategies.

Anecdotal reports from clinicians suggest that incidence of *Pneumocystis jirovecii* pneumonia, previously referred to as *P. carinii* pneumonia or PCP, among immunosuppressed patients, especially renal transplant recipients, has increased substantially (*1*). To investigate this claim, we analyzed data for January 2000 through December 2010, using several national data sources: Hospital Episode Statistics, routine laboratory reporting, death certificate data, and HIV surveillance data. 

*P. jirovecii* pneumonia gained notoriety during the AIDS pandemic (*2*); however, the reservoirs, modes of transmission, and pathogenesis of this organism remain poorly understood (*3*). Subclinical infection is considered common because studies have shown that anti–*P. jirovecii* antibodies develop during early childhood (*4*). Reactivation of latent infection after immunosuppression of the host was thought to be the main pathogenic mechanism (*3*); however, recent studies indicate that person-to-person spread might cause acute infection in susceptible persons (*5*).

Although not fully characterized, the known risk factors for *P. jirovecii* infection include impaired immunity because of HIV infection, hematologic malignancies, and connective tissue disorders (*6*). Immunosuppressive agents used to treat or prevent graft rejection have been implicated; such agents include corticosteroids, methotrexate, cyclosporine, mycophenolate mofetil, bendamustine, cyclophosphamide (*7*–*11*), and, recently, novel immunomodulating drugs, such as tumor necrosis factor–α inhibitors (*12*).

Prophylactically administered oral trimethoprim–sulfamethoxazole, dapsone, or atovaquone prevent the clinical manifestation of *P. jirovecii* infection. Also effective for decreasing *P. jirovecii* infection incidence among HIV-positive patients with a CD4^+^ count <200/μL is routine prophylactic administration of antimicrobial drugs (*13*,*14*).

Given the existence of effective chemoprophylaxis, identification of new risk groups might help prevent future increases in *P. jirovecii* infection incidence. Therefore, we conducted a retrospective analysis of multiple national data sources to examine trends in *P. jirovecii* infection. 

The Health Protection Agency has approval from the National Information Governance Board for Health and Social Care for the collation of surveillance data in accordance with section 251 of the National Health Service Act 2006. No additional ethical approval was required for this study.

## Materials and Methods

### Hospital Episode Statistics

The Hospital Episode Statistics (HES) database contains details of all inpatient admissions to National Health Service hospitals in England. We identified all patients for whom an International Classification of Diseases, 10th Revision (ICD-10), code B59, which corresponds with *P. jirovecii* infection, was recorded in any of the first 10 diagnosis fields from January 2000 through December 2010. By using ICD-10 and Operating Procedure Code Supplement 4 codes, we then subdivided cases into non–mutually exclusive, condition-specific categories that are frequently cited in the literature in association with *P. jirovecii* (*7*–*13*,*15*–*19*). The categories covered were renal failure, hematologic malignancy, other hematologic disorders, systemic connective tissue disorders, inflammatory diseases (such as rheumatoid or psoriatic arthritis), and receipt of immunosuppressive agents or an organ transplant. Patients with chronic lung conditions, such as pulmonary fibrosis, were categorized as a single group, given the observed frequency in this study of concurrence of this condition with *P. jirovecii* infection. Patients who did not fit into any risk category were also included in the analysis.

We cross-checked for duplicate records and selected the record of first admission for each patient. We examined information about sex, age, and geographic distribution of patients. HIV-infected patients were excluded from analysis because the clinical records for these patients did not contain patient-identifiable information (unlike the other clinical records in the HES database), thereby making identification and exclusion of duplicate records not possible for this group.

### Routine Laboratory Reporting

LabBase2 is the Health Protection Agency’s national communicable diseases database for England, Wales, and Northern Ireland; it receives semiautomated downloads of results from 99% of microbiology diagnostic laboratories (Health Protection Agency, unpub. data). Laboratory-confirmed cases of *P. jirovecii* infection in England during 2000–2010 were extracted from LabBase2, and duplicate laboratory samples were excluded.

### Death Certificate Data

For the study period, deaths in England with an ICD-10 clinical code indicating *P. jirovecii* as the cause or contributory cause of death were extracted from Office for National Statistics data. Deaths from *P. jirovecii* infection linked to a diagnosis of HIV or AIDS were also analyzed.

### HIV Surveillance Data

Data from the Health Protection Agency’s HIV and AIDS New Diagnoses and Deaths database were analyzed (*20*). Because HIV surveillance data are available for adults only, epidemiologic information in this study was restricted to patients >15 years of age. *P. jirovecii* infections were reported as co-infections at the time of HIV diagnosis, as subsequent AIDS diagnoses, or as the cause of death.

### Statistical Analyses

We used the statistical software STATA/SE 11.2 (*21*) for all analyses. Poisson regression with an offset for resident population, which used Office for National Statistics midyear estimates, was used to calculate the annual incidence rate ratio with 95% CIs. The Pearson χ^2^ test was used to examine changes in the proportion of cases by risk category over time (2000–2005 vs. 2006–2010).

## Results

**Figure 1:**
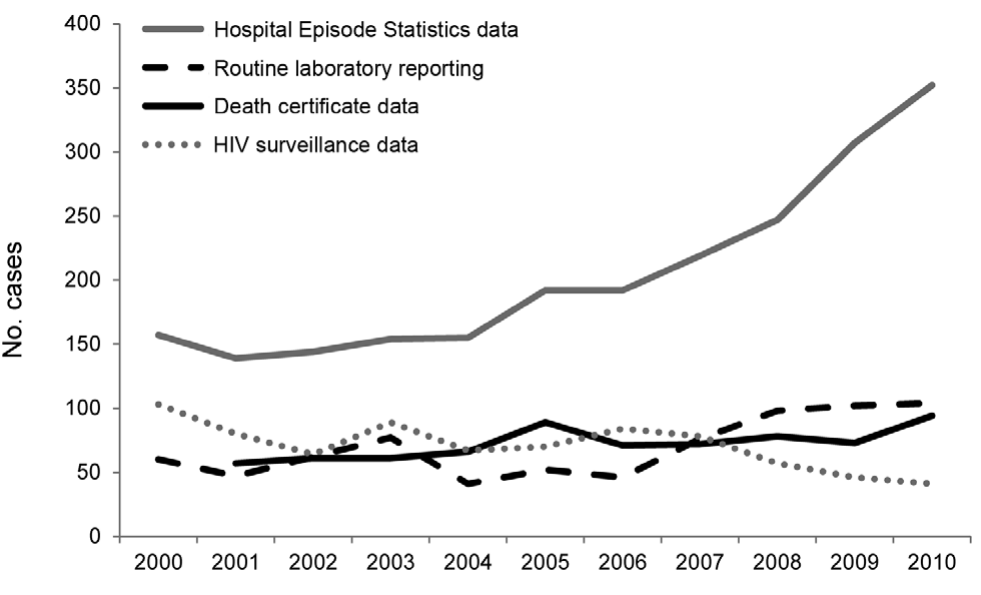
*Pneumocystis jirovecii* infections reported by national data collection systems, England, UK, 2000–2010. Hospital admissions exclude patients with HIV diagnoses.

The absolute numbers of cases of *P. jirovecii* pneumonia in England during 2000–2010, reported by each national surveillance system, are shown in Figure 1 and Table 1. We describe data from each system separately.

**Table 1:** Annual change in incidence rate of *Pneumocystis jirovecii* cases, England, UK, 2000–2010*

Surveillance system	Total no. cases	Annual incidence rate ratio (95% CI)†
Laboratory reporting	765	1.07 (1.05–1.09)
Hospital admissions‡	2,258	1.09 (1.08–1.11)
HIV surveillance data	779	0.94 (0.92–0.96)
Death registrations	722	1.04 (1.01–1.06)

*Midyear population estimates used.  †p<0.001 for all. ‡Excludes *P. jirovecii* diagnoses for patients with diagnosed HIV infection.

### Hospital Episode Statistics

During the study period, HES recorded 2,258 cases of *P. jirovecii* pneumonia. The number of cases increased from 157 in 2000 to 352 in 2010, an average annual increase of 9% (p<0.001).

**Figure 2:**
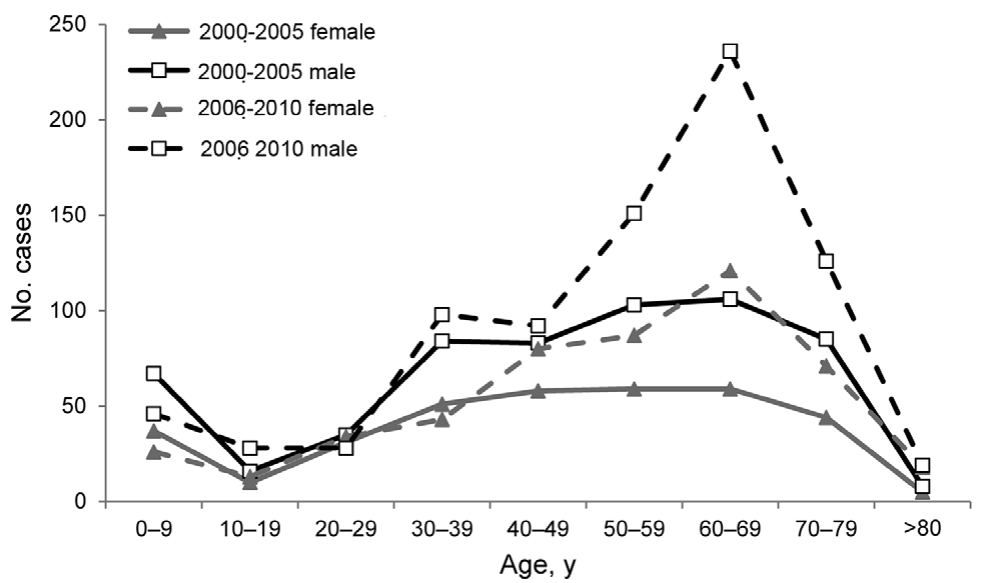
Age and sex distribution of patients with *Pneumocystis jirovecii* infections (excluding HIV-infected patients) among hospital admissions, England, UK, 2000–2010.

Cases reported to HES were not restricted to a particular geographic area, and the data showed no obvious seasonal trends. Because the increase in cases began in the latter half of the decade (Figure 1), we compared data from 2000–2005 with that from 2006–2010. This comparison showed a marked change in the age distribution of patients hospitalized for *P. jirovecii* infection during 2006–2010; relatively more patients were 60–69 years of age (Figure 2). Among all age groups, there was a higher proportion of male than female patients with *P. jirovecii* infection.

During the study period, 81% of patients within the HES database who had a diagnosis of *P. jirovecii* pneumonia could be classified according to a defined risk category (Table 2). Most (40.6%) had a hematologic malignancy, and 17.5% had preexisting lung disease. Relative distribution of risk groups differed significantly between 2000–2005 and 2006–2010 for all risk categories (χ^2^ 28.2, 7 degrees of freedom, p<0.001). The numbers of patients with *P. jirovecii* pneumonia increased significantly in all risk groups, but the difference in rates between the 2 periods was most marked among patients who had undergone transplantation, 47% of whom had undergone kidney transplantation during 2000–2010. The number of patients who were not in any of the risk groups described above dropped by 19% between the 2 periods. This test was conservative because there was some overlap between the risk categories.

**Table 2:** Proportion of all *Pneumocystis jirovecii*–associated hospital admissions and change in population rates over time, England, UK, 2000–2010

Risk category*	No. admissions (% all cases)			Annual rate/million population		Rate ratio between periods (95% CI)
	2000–2005	2006–2010		2000–2005	2006–2010	
Any transplant†	59 (6.3)	193 (14.7)		0.20	0.75	3.80 (2.84–5.09)
Other lung disease‡	120 (12.8)	276 (21.0)		0.24	0.47	1.97 (1.47–2.64)
Hematologic disorders	217 (23.1)	354 (26.9)		0.32	0.81	2.55 (2.00–3.25)
Hematologic malignancy	349 (37.1)	568 (43.1)		1.17	2.21	1.89 (1.66–2.16)
Connective tissue/inflammatory disease§	71 (7.6)	120 (9.1)		0.31	0.62	2.02 (1.56–2.61)
Renal failure and dialysis	95 (10.1)	208 (15.8)		0.16	0.35	2.23 (1.56–3.17)
Immunosuppressive/ chemotherapeutic drugs	47 (5.0)	90 (6.8)		0.73	1.38	1.90 (1.60–2.25)
Malignancy other than hematologic	92 (9.8)	160 (12.2)		0.40	1.07	2.67 (2.16–3.31)
Not in the above risk categories	255 (27.1)	177 (13.4)		0.85	0.69	0.81 (0.67–0.98)
Total no. cases¶	941	1,317		3.15	5.13	1.62 (1.50–1.77)

*Excludes HIV infection. †includes liver, heart, lung, kidney and bone transplants. ‡Includes tuberculosis, chronic obstructive pulmonary disease, cystic fibrosis, bronchiectasis, asthma, and interstitial lung disease. §Includes systemic connective tissue disorder, psoriatic arthropathy, rheumatoid arthritis, and inflammatory bowel disease. ¶Because some patients belong to >1 risk category, numbers do not add up to the total number of cases.

### Routine Laboratory Reporting

During the study period, LabBase2 recorded 765 laboratory-confirmed cases. Reported cases of *P. jirovecii* pneumonia remained relatively unchanged during 2000–2006 (range 41–77 cases/year, mean 55 cases/year) but increased from 76 cases in 2007 to 98–104 cases during 2008–2010 (Figure 1), particularly in older patients. The male-to-female ratio of *P. jirovecii* pneumonia patients during 2000–2010 was 2.5 to 1.0.

### Death Certificate Data

Deaths for which *P. jirovecii* pneumonia was recorded as a cause or contributing factor rose from 57 in 2001 to 94 in 2010 (p<0.001). For several years, the numbers of *P. jirovecii* infections reported on death certificates as a contributory cause of death were greater than those captured by laboratory reports (Figure 1).

### HIV Surveillance Data

**Figure 3:**
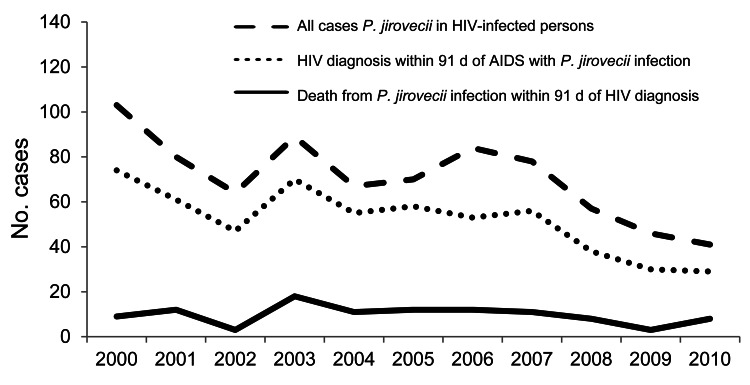
*Pneumocystis jirovecii* infections and deaths among persons with diagnosed HIV infection, England, UK, 2000–2010.

The numbers of patients with *P. jirovecii* pneumonia and HIV infection decreased 7% per year during 2000–2010 (p<0.001) (Figure 3). Most *P. jirovecii* infection diagnoses were made at the time of HIV diagnosis. Within this group of HIV-infected patients, death from *P. jirovecii* infection remained relatively stable over this period.

## Discussion

In this study, we found an increasing trend in rates for clinical cases recorded in HES and microbiologically confirmed and reported cases in England during 2000–2010. This finding suggests a real increase in the numbers of cases of *P. jirovecii* pneumonia diagnosed. We also found an association between *P. jirovecii* infection and a variety of chronic lung diseases not described in the literature as being associated with *P. jirovecii* infection. On the basis of these data, we propose preexisting lung disease as a new *P. jirovecii* pneumonia risk category.

The HES database yielded 2,258 cases of *P. jirovecii* pneumonia during 2000–2010, but LabBase2 found only 765. The differences in number of cases suggests substantial underreporting by laboratories, although most cases might be diagnosed on the basis of clinical or radiologic findings or by immunofluorescence in the cytology department without being microbiologically confirmed.

An analysis of the Health Protection Agency database of HIV-infected persons shows clear evidence of a substantial reduction in *P. jirovecii* infections during 2000–2010, consistent with an earlier diagnosis of HIV and receipt of effective antiretroviral therapy (*14*). *P. jirovecii* infections among HIV-infected persons declined, whereas *P. jirovecii* infections among non–HIV-infected persons increased, suggesting that other risk factors must be responsible for the increased numbers of cases.

Given the substantial illness and death associated with *P. jirovecii* infection and the resources needed to manage these cases, the increase in cases is of serious concern. Many patients need treatment in intensive care units. However, prophylactic use of antimicrobial drugs is highly effective for preventing the disease. A study in the United States suggested that almost $5 million a year could be saved in the state of Maryland alone if prophylaxis were instituted for all HIV-positive patients at risk for *P. jirovecii* infection (*22*).

### Potential Causes of the Observed Increase 

The increased number of cases might reflect changes in ascertainment of cases and increased infections in immunosuppressed patients who have received chemotherapy. It is possible that ascertainment increased over the study period because of improved diagnostic methods; immunofluorescence staining is being replaced by more sensitive PCR methods (*23*). We were not able to test the hypothesis that the increased number of cases is the result of increased testing for *P. jirovecii* because the laboratory surveillance system captures positive samples only, not the total number of samples submitted. However, the change in age distribution of patients toward a much older age group suggests that increased testing is not the main reason for increased case detection.

With regard to immunosuppression, an area that has seen an increase in the use of potent immunosuppressant agents is transplant surgery. Recipients who are not well matched to donor human leukocyte antigens now receive more powerful drugs. That said, the proportion of patients receiving renal transplants with a moderate degree of human leukocyte antigen mismatch has remained stable, represented by 43.9% of patients during financial year 2009–10 (National Health Service Blood and Transplant Authority, pers. comm.). Similarly, data from the National Health Service Blood and Transplant Authority indicate that the number of renal transplantations increased by 25% during 2006–2010. Again, this increase was not proportional to that observed for *P. jirovecii* infections reported for renal transplant recipients, which was ≈388% over the same period (National Health Service Blood and Transplant Authority, pers. comm.), so the increase cannot be explained simply by an increase in the number of patients in this risk group.

The largest group of persons affected by *P. jirovecii* pneumonia is those with hematologic malignancies. This finding might reflect the 30% increase in diagnoses of these malignancies during 2000–2010 (*24*). However, the increase in patients in this risk group with *P. jirovecii* pneumonia was 209% over the same period.

A possible explanation for the increase in *P. jirovecii* pneumonia cases is an increase in the number of potentially vulnerable patients who did not receive appropriate prophylactic therapy. Guidelines recommend the use of antimicrobial drug prophylaxis for kidney transplant recipients and for patients with hematologic malignancies who are receiving certain chemotherapy (*25*–*28*). A Cochrane review recommends prophylaxis for patients with hematologic malignancies and for recipients of bone marrow and solid organ transplants (*29*). Our study identified a new group at risk for *P. jirovecii* infection: patients with preexisting lung disease. To determine whether any preventative measures would be advisable for these patients will require further detailed characterization and quantification of risk within this group.

Another possible explanation for the increase in *P. jirovecii* pneumonia cases is increased transmission of the *P. jirovecii* organism between susceptible persons. Levels of exposure of susceptible persons to infectious persons might be increased as a result of changes in the delivery of health care. New, more transmissible strains could be emerging and leading to increased spread in the health care environment. Further investigation into the contribution of outbreaks—and, thus, increased person-to-person transmission—to the increase is warranted.

As a result of increased awareness of *P. jirovecii* infection, other infections might be clinically misdiagnosed as *P. jirovecii* infection. In the HES database, some patients might have been incorrectly coded as having *P. jirovecii* pneumonia, thereby resulting in a misclassification bias, but we have no reason to suspect that this coding would have changed over time. The death statistics should also be interpreted with caution because the cause of death and contributory causes are probably not recorded consistently. The analyses did not differentiate between outbreaks and sporadic cases of disease because this information could not be reliably determined from the data sources used. Although the most recent data might be subject to reporting delays, such delays would result in underestimation rather than overestimation of recent cases.

### Next Steps

Incidence of *P. jirovecii* pneumonia has increased across all groups of immunosuppressed patients known to be at risk for this infection (excluding HIV patients) and in new groups not previously known to be at risk. To determine whether current indications for prophylaxis need to be widened, enhanced surveillance should be introduced to help characterize any additional groups of patients for whom prophylaxis is not currently recommended but who might be at risk. Particular focus should be given to patients with chronic lung disease, systemic inflammatory diseases, and solid tumors and to transplant recipients who do not currently fulfill the criteria for prophylaxis. When introducing new immunosuppressive agents and regimens, consideration should be given as to whether these agents might increase the patients’ risk for *P. jirovecii* pneumonia.

More studies involving sequencing of *P. jirovecii* clinical isolates identified by PCR, coupled with national surveillance, should be used to better understand transmission dynamics and thereby inform infection control policies and clarify the role of any environmental factors (*1*,*30*–*32*). More basic knowledge of the biology, pathogenesis, virulence factors, and the contribution of different strains will be crucial for explaining observed changes in *P. jirovecii* epidemiology.

To ensure adherence to current guidelines and to ensure that preventive prophylaxis is optimal for all groups at risk for this potentially life-threatening infection, auditing of prescribing practices for patients known to be at risk is warranted. Raising awareness among clinicians could also help ensure that prophylaxis is correctly used.

In conclusion, data from a variety of national sources demonstrate an increase in the number of cases of *P. jirovecii* in non–HIV-infected persons. *P. jirovecii* infections are largely preventable by use of inexpensive drugs. The current case numbers are taking a substantial toll on health care costs and human health. Further investigation leading to improved preventive strategies for this largely preventable infection is warranted.
